# Impact of low cardiac function and diabetes mellitus on survival and causes of death following coronary artery surgery

**DOI:** 10.1093/icvts/ivaf144

**Published:** 2025-06-19

**Authors:** Sadayuki Moriyama, Akihiro Higashino, Yuya Tsuruta, Sumio Miura, Tsuyoshi Taketani, Minoru Ono, Takayuki Ohno

**Affiliations:** Department of Cardiovascular Surgery, Mitsui Memorial Hospital, Tokyo, Japan; Department of Cardiac Surgery, The University of Tokyo Hospital, Tokyo, Japan; Department of Cardiovascular Surgery, Mitsui Memorial Hospital, Tokyo, Japan; Department of Cardiovascular Surgery, Mitsui Memorial Hospital, Tokyo, Japan; Department of Cardiovascular Surgery, Mitsui Memorial Hospital, Tokyo, Japan; Department of Cardiovascular Surgery, Mitsui Memorial Hospital, Tokyo, Japan; Department of Cardiac Surgery, The University of Tokyo Hospital, Tokyo, Japan; Department of Cardiovascular Surgery, Mitsui Memorial Hospital, Tokyo, Japan

**Keywords:** coronary artery bypass grafting, low cardiac function, diabetes mellitus

## Abstract

**OBJECTIVES:**

To determine the differential impact of low cardiac function (ejection fraction [EF] ≤ 35%) and diabetes mellitus (DM) on survival and to identify causes of death after coronary artery bypass grafting (CABG).

**METHODS:**

Overall, 1036 patients who underwent isolated CABG between 2009 and 2022 were divided into four groups based on EF and DM. Kaplan–Meier analysis was performed to calculate each group’s estimated survival. Inter-group multivariate Cox regression was performed with the reference group showing EF > 35% and DM (−). Additional Cox regressions were performed to investigate the associations of EF ≤ 35% and DM (+) with death from heart failure, myocardial infarction, cancer, pneumonia, cerebrovascular disease and renal failure.

**RESULTS:**

Off-pump techniques were used in 980 patients (95%). Patient population and estimated 10-year postoperative survival were as follows: EF > 35% DM (−), 430, 75.1%; EF > 35% DM (+), 456, 66.3%; EF ≤ 35% DM (−), 73, 62.5%; and EF ≤ 35% DM (+), 77, 53.5%. Hazard ratios (HRs) (*P* values) for the three groups were as follows: EF > 35% DM (+), 1.53 (0.006); EF ≤ 35% DM (−), 1.84 (0.017); and EF ≤ 35% DM (+), 2.23 (0.001). For death from heart failure, HR (*P* value) for EF ≤ 35% versus EF > 35% was 3.62 (0.012). For deaths from cancer and pneumonia, HRs (*P* values) for DM (+) versus DM (−) were 1.73 (0.097), and 2.72 (0.046), respectively.

**CONCLUSIONS:**

EF ≤ 35% and DM (+) are associated with worse post-CABG survival. Each is associated with specific causes of death.

## GRAPHICAL ABSTRACT

**Figure:**
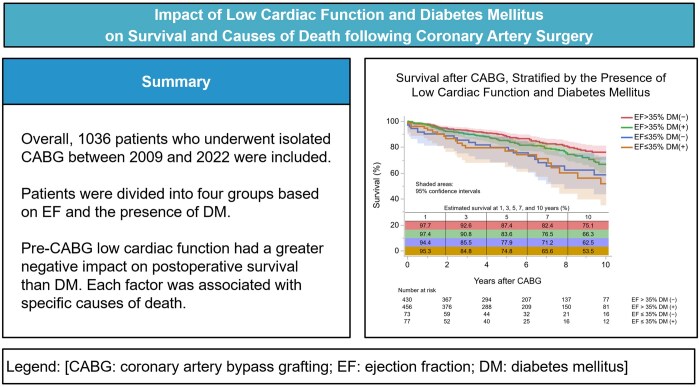

## INTRODUCTION

Coronary artery bypass grafting (CABG) provides better mid- to long-term survival than other treatments, depending on the underlying disease. Higher survival was reported in the CABG plus medical therapy group than in the medical therapy-only group for patients with lower cardiac function (ejection fraction [EF] ≤ 35%) [1]. In some studies, CABG resulted in better survival than percutaneous coronary intervention (PCI) in patients with low cardiac function [2, 3] and PCI with drug-eluting stents in patients with diabetes mellitus (DM) and multivessel coronary artery disease [4]. Low cardiac function and DM are important prognostic factors after CABG. However, few studies have examined their combined impact on post-CABG outcomes. Identifying specific causes of death in patients with low cardiac function and DM following CABG could further improve survival. Therefore, we investigated their impact on survival and causes of death following CABG.

## PATIENTS AND METHODS

### Ethics statement

This study complies with the principles of the Declaration of Helsinki. The Institutional Review Board approved this retrospective study and waived written informed consent (C95, 11 March 2024).

### Study design

Patients who underwent isolated CABG between April 2009 and December 2022 at Mitsui Memorial Hospital, Tokyo, Japan, were retrospectively studied. Exclusion criteria were no echocardiography within 6 months preoperatively, CABG for postoperative complications and re-CABG.

### Surgery and follow-up

CABG strategy involved a median sternotomy and off-pump techniques, with on-pump techniques used in cases of unstable circulation. Left internal thoracic artery anastomosis was preferred for the left anterior descending artery and right internal thoracic artery anastomosis for the diagonal branch, proximal left circumflex artery and obtuse marginal branch. We mainly used the great saphenous vein for the distal left circumflex and right coronary arteries.

After discharge, medications were adjusted for patients referred from other hospitals at their respective referring hospitals. Each patient referred from our hospital’s cardiology department was examined and had their medication adjusted while at the cardiology department. Once a patient’s condition was stabilised, the prescription was continued by their general practitioner. The cardiovascular surgery department follow-up schedule was as follows: 1–2 weeks after discharge, at 2 months and every 3 months thereafter, with adjustments made based on the patient’s condition.

### Statistical analysis

#### Interaction between EF and DM, baseline characteristics, operative data, discharge medications and insulin use as a risk factor

We first assessed the interaction between cardiac function (EF ≤ 35%, > 35%) and DM using the χ^2^ test. The patients were then divided into four groups based on the presence or absence of EF ≤ 35% and DM. Continuous variables were assessed for normality by checking histograms and performing Q–Q plots. Categorical variables were examined for the corresponding number and percentage of persons in each group or the overall population. Patients with EF > 35% and non-DM, considered the healthiest, were tested against other groups as the reference group. To prevent issues with multiple comparisons, we applied Dunnett’s and Steel’s tests for normally and non-normally distributed data, respectively. *P* values (significance level 0.05) were calculated for each test. Categorical variables were compared between the reference group and each of the other groups using the χ^2^ test. If one or more expected frequencies were ≤10, *P* values were calculated using two-tailed Fisher’s exact tests. Each *P* value was assessed for significance using the Holm–Bonferroni method [5]. For the smallest *P* value among the three tests, the significance level was set at 0.0166 (0.05 divided by the number of tests). If this *P* value was larger, no conclusions were drawn for the tests. If smaller, it was considered significant, and the next smallest *P* value was assessed. The significance level was then adjusted to 0.025 (0.05 divided by the number of tests minus 1). If this *P* value was larger, conclusions were withheld for this test and the one with the largest *P* value. If smaller, it was considered significant, and the test with the largest *P* value was reviewed. The final significance level was set at 0.05. If this level was exceeded, no conclusions were drawn; if not, the test was considered significant. If one or two of the three tests could not be performed, they were excluded, and the significance level was adjusted accordingly. EF data were collected within 6 months preoperatively. The measurement methods were Simpson’s method (biplane), Simpson’s method (monoplane) and the Teichholz method, in that order of preference. If multiple echocardiographic examinations were performed using the same method, the lowest measured values were used. DM was defined by one or more of the following conditions: the most recent glycated haemoglobin ≥ 6.5% on blood tests within 6 months preoperatively, regular perioperative use of oral hypoglycaemic agents (excluding sodium–glucose cotransporter 2 [SGLT2] inhibitors used solely for heart failure), or regular use of subcutaneous insulin injections. Insulin use as a risk factor was investigated as described in the Supplementary Method S1.

#### Primary outcomes: post-CABG survival and hazard ratios for four groups divided by the presence of EF ≤ 35% and DM

Survival status as of 1 April 2023 was assessed through medical records or telephonic confirmation with patients or relatives. Death from any cause was considered an event, and the time from surgery to death was analysed. Patients alive or lost to follow-up were censored. Kaplan–Meier analysis was performed for the four groups based on EF ≤ 35% and DM. Estimated survival and standard errors at 1, 3, 5, 7 and 10 years postoperatively were calculated, assuming a Weibull distribution. To examine the effect of EF ≤ 35% and DM on post-CABG survival, we performed multivariate Cox regression adjusted for covariates including sex, age at surgery ≥ 75 years, body mass index ≥ 30, preoperative dialysis, smoking status (current, former or never) and distal arterial anastomoses ≥ 2, based on previous literature [6, 7]. Hazard ratios (HRs), confidence intervals (CIs), and *P* values (significance level 0.05) were calculated with EF > 35% and non-DM as the reference group.

#### Secondary outcomes: HRs of EF ≤ 35% versus EF > 35%, and DM versus non-DM by causes of death

Causes of death during follow-up were investigated using death certificates or telephonic confirmation. The prevalence of causes of death (heart failure, myocardial infarction, cancer, pneumonia, cerebrovascular disease and renal failure) was examined for EF ≤ 35% and DM. Renal failure deaths were those caused by dysdialysis or lethal arrhythmias due to hyperkalaemia. Multivariate Cox regressions adjusted for covariates (sex, age at surgery ≥ 75 years, body mass index ≥ 30, preoperative dialysis, smoking status and distal arterial anastomoses ≥ 2) were performed for EF ≤ 35% versus EF > 35%, and for DM versus non-DM, considering each cause of death.

All statistical analyses were performed using JMP Pro 17.2.0 (SAS Institute, Cary, NC, USA).

## RESULTS

From April 2009 to December 2022, 1063 patients underwent isolated CABG at our institution, and 1036 were included after excluding 27 patients. Of the 1036 patients, 65 were lost to follow-up, and their outcomes were unavailable in the telephonic confirmation. The χ^2^ test result for the presence of EF (≤ 35%, > 35%) and DM was *P *=* *0.98, indicating no interaction between DM and EF.

Table 1 shows baseline characteristics. Of the included patients, 430 had EF > 35% and non-DM, 456 had EF > 35% and DM, 73 had EF ≤ 35% and non-DM, and 77 had EF ≤ 35% and DM. The mean age was 67.8 years, and the median follow-up period was 5.4 years. No significant between-group differences in ischaemic lesions, proportion of stable angina, unstable angina, myocardial infarction and preoperative dialysis rates were observed.

**Table 1:** Baseline characteristics^a^

	EF > 35%, DM (−)	EF > 35%, DM (+)	EF ≤ 35%, DM (−)	EF ≤ 35%, DM (+)	Overall	*P* value (reference: EF > 35%, DM (−))		
Characteristic	*n* = 430	*n* = 456	*n* = 73	*n* = 77	*N* = 1036	EF > 35%, DM (+)	EF ≤ 35%, DM (−)	EF ≤ 35%, DM (+)
Age at surgery, years (SD)	68.6 (10.5)	67.8 (9.2)	65.8 (11.3)	65.1 (10.7)	67.8 (10.1)	0.51	0.074	**0.013**
Age at surgery ≥ 75 years, *n* (%)	143 (33)	121 (27)	16 (22)	13 (17)	293 (28)	0.029	0.054	**0.004**
Female, *n* (%)	68 (16)	64 (14)	4 (5.5)	8 (10)	144 (14)	0.46	0.020	0.22
Follow-up period, years (IQR)	5.8 (3.3–8.8)	5.4 (2.8–8.8)	5.3 (2.3–8.9)	4.1 (1.3–7.2)	5.4 (2.8–8.8)	0.62	0.49	**0.005**
Ejection fraction, % (IQR)	62 (54–67)	60 (51–67)	27 (22–32)	28 (21–33)	59 (45–66)	**0.027**	**< 0.001**	**< 0.001**
Oral hypoglycaemic agent, *n* (%)	1 (0.2)	314 (69)	4 (5.5)	50 (65)	369 (36)	**< 0.001**	**0.002**	**< 0.001**
Insulin use, *n* (%)	0	173 (38)	0	33 (43)	206 (20)	**< 0.001**	N/A	**< 0.001**
HbA1c, % (IQR)	5.7 (5.5–6.0), n = 424	7.1 (6.7–8.0), n = 452	5.8 (5.5–6.0), n = 72	7.4 (6.9–8.4), n = 77	6.3 (5.7–7.2)	**< 0.001**	0.79	**< 0.001**
Type 1 DM, *n* (%)	0	7 (1.5)	0	1 (1.3)	8 (0.8)	**0.016**	N/A	0.15
BMI, kg/m^2^ (SD)	22.7 (3.3)	23.8 (3.7)	22.3 (4.0)	23.1 (4.8)	23.2 (3.7)	**< 0.001**	0.82	0.72
BMI ≥ 30 kg/m^2^, *n* (%)	9 (2.1)	26 (5.7)	4 (5.5)	9 (12)	48 (4.6)	**0.006**	0.10	**< 0.001**
Smoking status, *n* (%)								
Current	78 (18)	83 (18)	18 (25)	22 (29)	201 (19)	0.61	0.36	0.10
Former	213 (50)	239 (53)	33 (46)	35 (45)	520 (50)			
Never	138 (32)	133 (29)	20 (28)	20 (26)	311 (30)			
Preoperative dialysis, *n* (%)	61 (14)	54 (12)	9 (12)	19 (25)	143 (14)	0.30	0.67	0.020
Stable angina, *n* (%)	332 (77)	352 (77)	60 (82)	60 (78)	804 (78)	1.00	0.34	0.89
Unstable angina, *n* (%)	60 (14)	65 (14)	4 (5.5)	8 (10)	137 (13)	0.90	0.055	0.40
Myocardial infarction, *n* (%)	38 (8.8)	39 (8.6)	9 (12)	9 (12)	95 (9.2)	0.88	0.38	0.40
Diseased coronary arteries, *n* (%)								
One branch	23 (5.4)	16 (3.5)	4 (5.5)	0	43 (4.2)	0.18	1.00	0.035
Two branches	101 (23)	82 (18)	18 (25)	11 (14)	212 (20)	0.043	0.83	0.073
Three branches	191 (44)	223 (49)	32 (44)	42 (55)	488 (47)	0.18	0.93	0.10
Only LMT	7 (1.6)	2 (0.4)	1 (1.4)	0	10 (1.0)	0.099	1.00	0.60
LMT + one branch	13 (3.0)	13 (2.9)	2 (2.7)	1 (1.3)	29 (2.8)	0.88	1.00	0.71
LMT + two branches	45 (10)	44 (9.7)	6 (8.2)	10 (13)	105 (10)	0.69	0.68	0.55
LMT + three branches	50 (12)	76 (17)	10 (14)	13 (17)	149 (14)	0.032	0.56	0.19

a *P* values were calculated by comparing the reference group (EF > 35%, DM (−)) with each of the following groups: EF > 35%, DM (+); EF ≤ 35%, DM (−); and EF ≤ 35%, DM (+). Dunnett’s tests were used for normally distributed continuous variables, Steel’s tests for non-normally distributed continuous variables, and either χ^2^ or two-tailed Fisher’s exact tests for categorical variables. Significance levels of categorical variables were adjusted using the Holm–Bonferroni method. Bolded *P* values indicate statistical significance.

BMI, body mass index; DM, diabetes mellitus; EF, ejection fraction; IQR, interquartile range; LMT, left main trunk; SD, standard deviation; SGLT2, sodium–glucose cotransporter 2.

Table 2 shows operative data: 95% of surgeries were off-pump, with an average of 3.4 anastomoses. The EF ≤ 35% groups had significantly fewer off-pump surgeries and more anastomoses than the reference group.

**Table 2:** Operative data^a^

	EF > 35%, DM (−)	EF > 35%, DM (+)	EF ≤ 35%, DM (−)	EF ≤ 35%, DM (+)	Overall	*P* value (reference: EF > 35%, DM (−))		
Variable	*n* = 430	*n* = 456	*n* = 73	*n* = 77	*N* = 1036	EF > 35%, DM (+)	EF ≤ 35%, DM (−)	EF ≤ 35%, DM (+)
Off-pump, *n* (%)	420 (98)	444 (97)	52 (71)	64 (83)	980 (95)	0.77	**< 0.001**	**< 0.001**
Use of ITA, *n* (%)	421 (98)	450 (99)	70 (96)	77 (100)	1018 (98)	0.44	0.40	0.37
BITA	298 (69)	326 (71)	40 (55)	56 (73)	720 (69)	0.48	**0.015**	0.55
LITA	102(24)	108 (24)	27 (37)	19 (25)	256 (25)	0.99	**0.016**	0.86
RITA	21 (4.9)	16 (3.5)	3 (4.1)	2 (2.6)	42 (4.1)	0.31	1.00	0.55
Use of RA, *n* (%)	8 (1.9)	5 (1.1)	2 (2.7)	1 (1.3)	16 (1.5)	0.41	0.64	1.00
Use of GEA, *n* (%)	6 (1.4)	8 (1.8)	1 (1.4)	3 (3.9)	18 (1.7)	0.79	1.00	0.14
Anastomoses, *n* (SD)	3.3 (1.0)	3.4 (1.0)	3.7 (1.2)	3.8 (1.0)	3.4 (1.0)	0.20	**0.002**	**< 0.001**
DAA, *n* (SD)	1.7 (0.5)	1.7 (0.5)	1.5 (0.6)	1.8 (0.5)	1.7 (0.5)	0.92	0.058	0.68
DAA ≥ 2, *n* (%)	304 (71)	330 (72)	41 (56)	58 (75)	733 (71)	0.58	**0.013**	0.41

a *P* values were calculated by comparing the reference group (EF > 35%, DM (−)) with each of the following groups: EF > 35%, DM (+); EF ≤ 35%, DM (−); and EF ≤ 35%, DM (+). Dunnett’s tests were used for normally distributed continuous variables and either χ^2^ or two-tailed Fisher’s exact tests for categorical variables. Significance levels of categorical variables were adjusted using the Holm–Bonferroni method. Bolded *P* values indicate statistical significance.

BITA, bilateral thoracic artery; DAA, distal arterial anastomoses; DM, diabetes mellitus; EF, ejection fraction; GEA, gastroepiploic artery; ITA, internal thoracic artery; LITA, left internal thoracic artery; RA, radial artery; RITA, right internal thoracic artery; SD, standard deviation.

Table 3 shows discharge medications. Patients with EF ≤ 35% were significantly more likely to take β-blockers than the reference group. The other three groups were significantly more likely to take SGLT2 inhibitors than the reference group, although the number of patients receiving SGLT2 inhibitors was relatively small.

**Table 3:** Discharge medications^a^

	EF > 35%, DM (−) *n* = 427	EF > 35%, DM (+) *n* = 453	EF ≤ 35%, DM (−) *n* = 70	EF ≤ 35%, DM (+) *n* = 75	Overall *n* = 1025	*P* value (reference: EF > 35%, DM (−))		
Medication	*n* (%)	*n* (%)	*n* (%)	*n* (%)	*n* (%)	EF > 35%, DM (+)	EF ≤ 35%, DM (−)	EF ≤ 35%, DM (+)
Calcium blocker	108 (25)	117 (26)	10 (14)	11 (15)	246 (24)	0.86	0.045	0.046
ACEI	30 (7.0)	30 (6.6)	11 (16)	8 (11)	79 (7.7)	0.81	0.031	0.34
ARB	51 (12)	60 (13)	4 (5.7)	8 (11)	123 (12)	0.56	0.15	0.85
β-blocker	262 (61)	295 (65)	58 (83)	60 (80)	675 (66)	0.25	**< 0.001**	**0.002**
Statin	374 (88)	394 (87)	59 (84)	61 (81)	888 (87)	0.79	0.44	0.14
ARNI	1 (0.2)	2 (0.4)	2 (2.9)	2 (2.7)	7 (0.7)	1.00	0.053	0.060
SGLT2 inhibitor	1 (0.2)	53 (12)	4 (5.7)	19 (25)	77 (7.5)	**< 0.001**	**0.002**	**< 0.001**
Aspirin	398 (93)	432 (95)	66 (94)	70 (93)	966 (94)	0.17	1.00	1.00
P2Y12 inhibitor	74 (17)	62 (14)	10 (14)	8 (11)	154 (15)	0.14	0.53	0.15

a *P* values were calculated by comparing the reference group (EF > 35%, DM (−)) with each of the following groups: EF > 35%, DM (+); EF ≤ 35%, DM (−); and EF ≤ 35%, DM (+). χ^2^ or two-tailed Fisher’s exact tests were conducted. Significance levels were adjusted using the Holm–Bonferroni method. Bolded *P* values indicate statistical significance.

ACEI, angiotensin-converting enzyme inhibitor; ARB, angiotensin II receptor blocker; ARNI, angiotensin receptor-neprilysin inhibitor; DM, diabetes mellitus; EF, ejection fraction; SGLT2, sodium–glucose cotransporter 2.

Supplementary Figure S1 and Supplementary Table S1 demonstrate that patients with insulin-dependent DM have slightly lower survival compared with those with non-insulin-dependent DM and non-DM patients. However, in the multivariable Cox regression, both insulin-dependent and non-insulin-dependent DM were associated with similar HRs compared with non-DM patients: 1.44 (95% CI: 1.03–2.01, *P *=* *0.031) and 1.48 (95% CI: 1.08–2.03, *P *=* *0.015), respectively.

**Figure 1:**
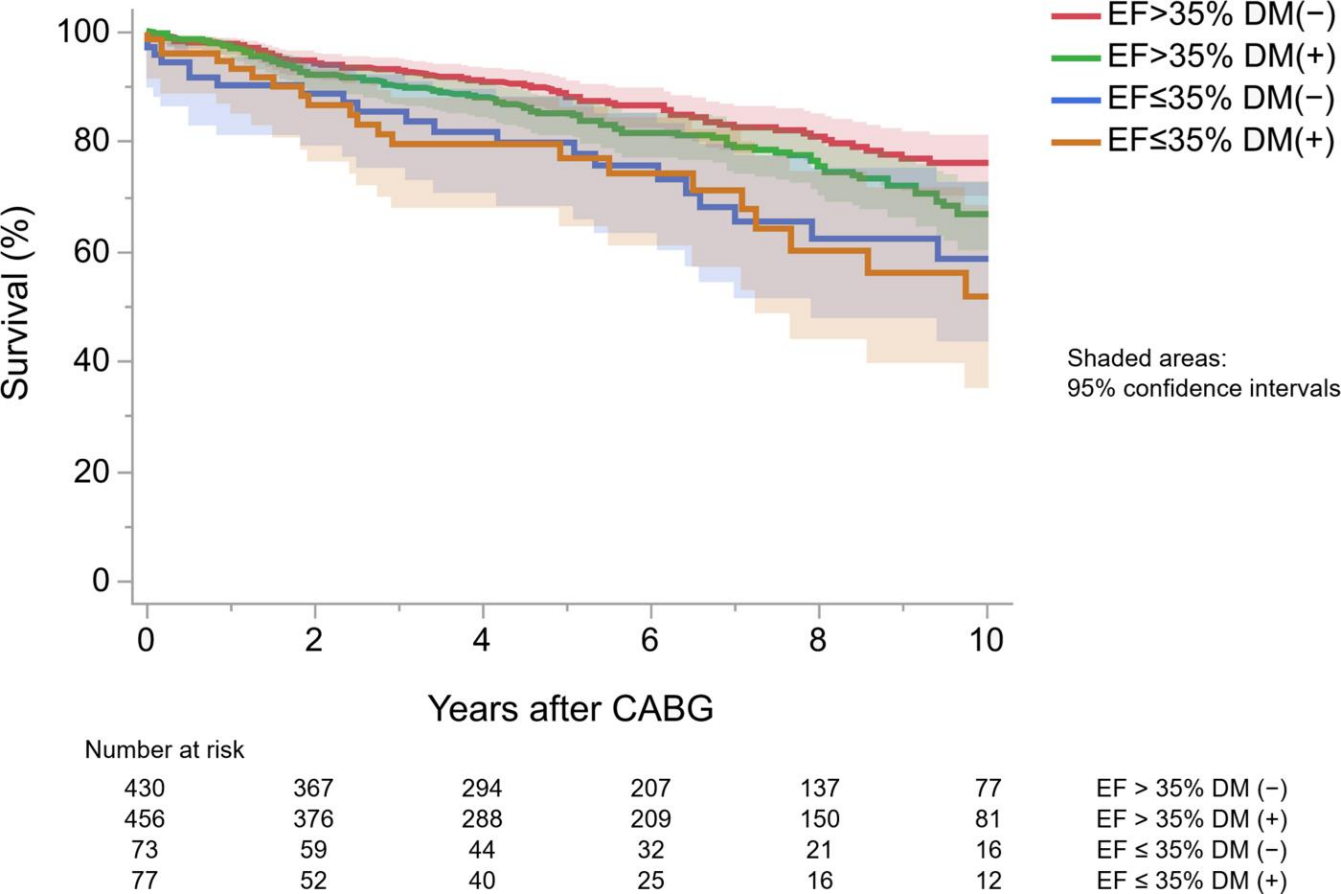
Kaplan–Meier analysis for patients who underwent coronary artery bypass grafting, stratified by the presence of low cardiac function and diabetes mellitus. CABG, coronary artery bypass grafting; DM, diabetes mellitus; EF, ejection fraction

Figure 1 shows Kaplan–Meier analysis results: patients with EF > 35% with non-DM (red), EF > 35% with DM (blue), EF ≤ 35% with non-DM (green) and EF ≤ 35% with DM (ochre). Table 4 shows each group’s estimated survival, with a 21.6% difference in 10-year survival between patient groups with EF > 35% and DM (−), and those with EF ≤ 35% and DM (+).

**Table 4:** Estimated survival after coronary artery bypass grafting

	1 year	3 years	5 years	7 years	10 years
Group	Survival (%) (SE)	Survival (%) (SE)	Survival (%) (SE)	Survival (%) (SE)	Survival (%) (SE)
EF > 35%, DM (−)	97.7 (0.6)	92.6 (1.1)	87.4 (1.5)	82.4 (1.9)	75.1 (2.6)
EF > 35%, DM (+)	97.4 (0.6)	90.8 (1.2)	83.6 (1.6)	76.5 (2.0)	66.3 (2.7)
EF ≤ 35%, DM (−)	94.4 (2.3)	85.5 (3.7)	77.9 (4.6)	71.2 (5.4)	62.5 (6.8)
EF ≤ 35%, DM (+)	95.3 (2.0)	84.8 (3.7)	74.8 (4.8)	65.6 (5.9)	53.5 (7.5)

DM, diabetes mellitus; EF, ejection fraction; SE, standard error.

Table 5 shows adjusted Cox regression results for mortality following CABG. HRs were 1.53 for patients with EF > 35% and DM, 1.84 for patients with EF ≤ 35% and non-DM, and 2.23 for patients with EF ≤ 35% and DM. All results were statistically significant. EF ≤ 35% was associated with a worse prognosis than DM.

**Table 5:** Adjusted Cox regression for mortality following coronary artery bypass grafting^a^

Group (reference: EF > 35%, DM (−))	Hazard ratio	95% confidence interval	*P* value
EF > 35%, DM (+)	1.53	1.13–2.06	**0.006**
EF ≤ 35%, DM (−)	1.84	1.12–3.05	**0.017**
EF ≤ 35%, DM (+)	2.23	1.38–3.60	**0.001**

a Covariates of Cox regression: sex, age at surgery ≥ 75, body mass index ≥ 30, preoperative dialysis, smoking status (current, former, never) and distal arterial anastomoses ≥ 2. Bolded *P* values indicate statistical significance.

DM, diabetes mellitus; EF, ejection fraction.

Table 6 shows adjusted Cox regression results for causes of death following CABG. EF ≤ 35% was only associated with death from heart failure compared with EF > 35%. DM was significantly associated with pneumonia-related death compared with non-DM. Cancer-related death was not a statistically significant cause of mortality associated with DM compared with non-DM, but a trend was observed.

**Table 6:** Adjusted Cox regressions for causes of death following coronary artery bypass grafting^a^

		EF ≤ 35% (reference: EF > 35%)			DM (+) (reference: DM (−))		
Cause of death	Deaths, *n*	Hazard ratio	95% confidence interval	*P* value	Hazard ratio	95% confidence interval	*P* value
Overall	226						
Heart failure	19	3.62	1.32–9.87	**0.012**	0.96	0.38–2.41	0.92
Myocardial infarction	9	3.26	0.59–18.09	0.18	1.53	0.40–5.86	0.53
Cancer	40	0.55	0.17–1.80	0.32	1.73	0.91–3.32	0.097
Pneumonia	19	2.19	0.71–6.76	0.17	2.72	1.02–7.29	**0.046**
Cerebrovascular disease	11	2.60	0.67–10.07	0.17	2.75	0.71–10.60	0.14
Renal failure	13	1.92	0.50–7.28	0.34	0.90	0.29–2.81	0.86
Deaths from unknown causes	66	1.57	0.84–2.92	0.16	1.19	0.72–1.95	0.50

a Covariates of Cox regressions: female sex, age at surgery ≥ 75, body mass index ≥ 30, preoperative dialysis, smoking status (current, former, never), and distal arterial anastomoses ≥ 2. Bolded *P* values indicate statistical significance.

DM, diabetes mellitus; EF, ejection fraction.

## DISCUSSION

Previous studies have demonstrated differences in survival after CABG by focusing on either cardiac function or DM. This study revealed survival differences based on both cardiac function and DM. Patients with EF ≤ 35% or DM, or both, had worse outcomes than those with EF > 35% and non-DM. In particular, the 21.6% difference in 10-year survival between patient groups with EF > 35% and DM (−) (75.1%) and those with EF ≤ 35% and DM (+) (53.5%) helped us understand the prognostic difference. Our study is the first to demonstrate this result. HRs were calculated to quantify the impact of EF ≤ 35% and DM on outcomes following CABG. EF ≤ 35% was only associated with death from heart failure. DM was related to cancer- and pneumonia-related deaths.

Previous studies [8–10] have shown that low cardiac function is associated with lower post-CABG survival. Although different criteria were used to define low and preserved cardiac function, all studies demonstrated a sharp decline in survival within the first postoperative year in the low cardiac function group, followed by an earlier drop in survival compared with the preserved function group. Improvements in surgical technique and perioperative management might improve early mortality. Survival decline from 1 to 10 years may suggest that low cardiac function is disadvantageous for patients in the mid- and long-term postoperative period. Few studies have investigated the causes of death following CABG according to preoperative low cardiac function versus preserved cardiac function. This study demonstrated the effect of low cardiac function versus preserved cardiac function on death from heart failure. Low cardiac function is a reduction in the heart’s pumping power and may lead to cardiovascular death [11]. Medical therapy might help reduce or delay these deaths.

One report [12] found no significant difference in post-CABG survival between patients with DM and those without DM, while others [13–15] found the opposite. In this study, DM was associated with a significantly reduced survival among patients with EF > 35% (Fig. 1 and Table 5). Among patients with EF ≤ 35%, DM correlated with lower survival. Whether the difference in survival was due to specific causes of death should be considered. A report [16] found no significant difference in cardiac death up to 10 years postoperatively between patients with DM and non-DM without comorbidities such as low cardiac function, consistent with our findings. We also investigated other causes of death, and pneumonia-related death after CABG was significantly different between patients with DM and those without. There was also a trend towards the association of cancer-related death in DM compared with non-DM. Cancer and pneumonia were also noted as common causes of death in DM [17], and the presence of DM has been pathologically associated with increased susceptibility to cancer [18] and to pneumonia [19]. Survival differences by the presence of DM could be clinically explained as deaths from DM-related conditions rather than as vascular transformation associated with hyperglycaemia.

A study [20] found that CABG was superior to PCI in terms of survival and complications following CABG and PCI in patients with both low cardiac function and DM. However, even with CABG, survival remains poor in the group. Table 4 shows a 21.6% difference in 10-year postoperative survival between EF > 35% DM (−) and EF ≤ 35% DM (+). This study raised the issue of resolving cause-of-death biases to improve postoperative survival in patients with low cardiac function and DM.

This study has several limitations. First, it is a single-centre, retrospective study from Japan, which may limit generalisability owing to selection bias. Additionally, the small sample size with low cardiac function may have affected statistical power. Echocardiography lacks a unified method for measuring EF, which introduces bias in cardiac function assessment. Increase or decrease in discharge medications during follow-up may affect mid- and long-term survival. Causes of death obtained from relatives and unknown causes of death also present limitations.

## CONCLUSIONS

Patients with low cardiac function or DM had worse outcomes than those without. Low cardiac function was associated with higher mortality only from heart failure during follow-up. DM showed a trend towards cancer- and pneumonia-related deaths.

## Supplementary Material

ivaf144_Supplementary_Data

## Data Availability

The data underlying this paper will be shared by the corresponding author upon reasonable request.

## ABBREVIATIONS

CABG: Coronary artery bypass grafting
CI: Confidence interval
DM: Diabetes mellitus
EF: Ejection fraction
HR: Hazard ratio
PCI: Percutaneous coronary intervention
SGLT2: Sodium–glucose cotransporter 2
