# Supplementary material for: Impact of low cardiac function and diabetes mellitus on survival and causes of death following coronary artery surgery
Source: Interdiscip Cardiovasc Thorac Surg. 2025 Jun 19;40(7):ivaf144. doi: 10.1093/icvts/ivaf144 (PMC12231534; doi:10.1093/icvts/ivaf144)
Supplement: ivaf144_Supplementary_Data [file ivaf144_supplementary_data.docx]

Supplementary Materials

Method S1. We investigated the survival of patients with diabetes mellitus (DM) based on insulin dependency and whether insulin dependency itself was a risk factor. Patients were divided into three groups: non-DM, non-insulin-dependent DM, and insulin-dependent DM. Kaplan–Meier analysis was performed. Estimated survival and standard errors at 1, 3, 5, 7, and 10 years postoperatively were calculated, assuming a Weibull distribution. Multivariable Cox regression was performed to evaluate whether insulin dependency was a risk factor compared with non-DM. Covariates included female sex, age at surgery ≥ 75, body mass index ≥ 30, preoperative dialysis, smoking status (current, former, never), distal arterial anastomoses ≥ 2, and ejection fraction (≤ 35%, > 35%).

Figure S1. Kaplan–Meier analysis for patients who underwent coronary artery bypass grafting, stratified by diabetes mellitus status: non-diabetes mellitus, non-insulin-dependent, and insulin-dependent diabetes mellitus
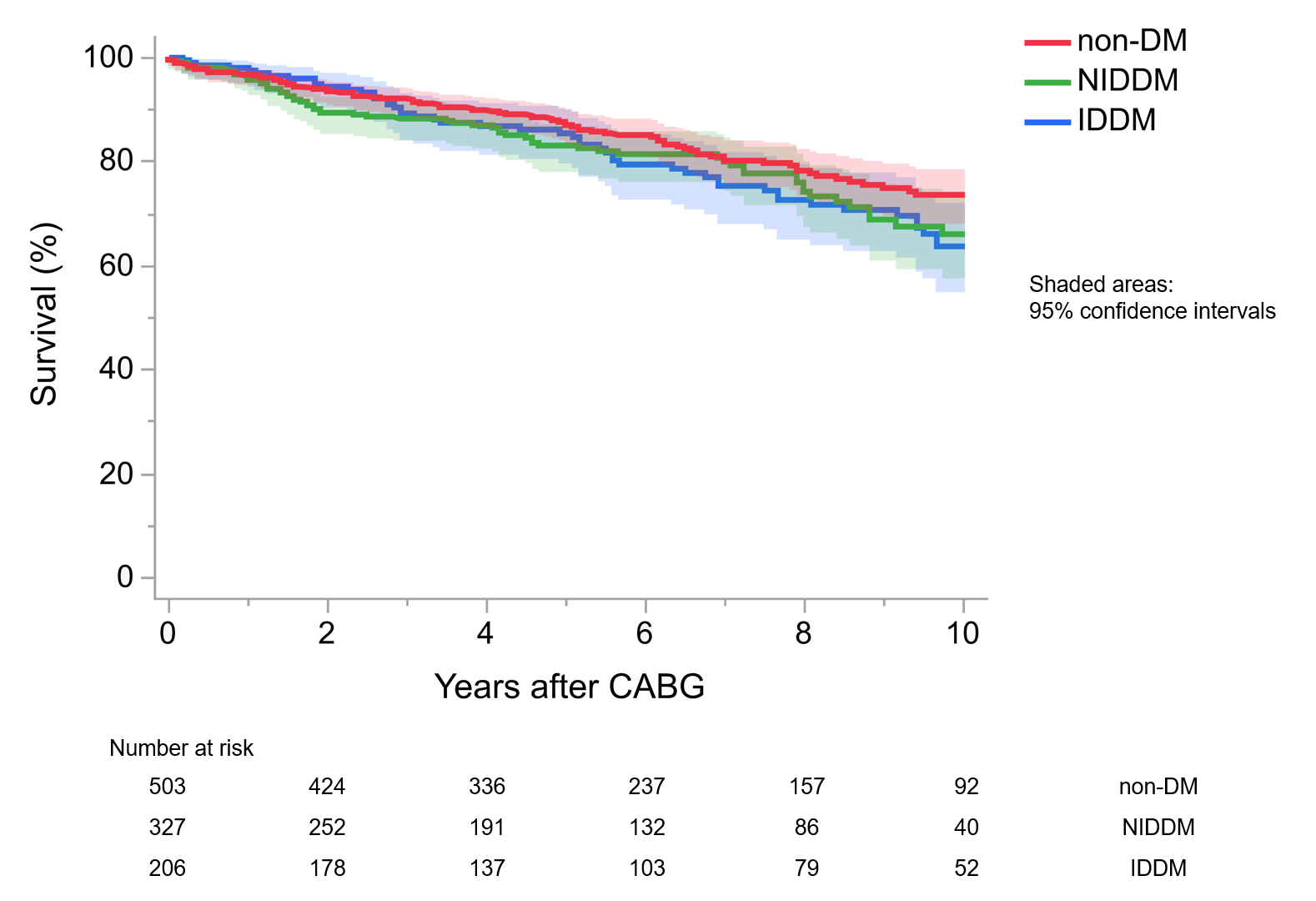


CABG, coronary artery bypass grafting; non-DM, non-diabetes mellitus; NIDDM, non-insulin-dependent diabetes mellitus; IDDM, insulin-dependent diabetes mellitus

Table S1. Estimated survival after coronary artery bypass grafting, stratified by diabetes mellitus status: non-diabetes mellitus, non-insulin-dependent, and insulin-dependent diabetes mellitus

|  | 1 year | 3 years | 5 years | 7 years | 10 years |
| --- | --- | --- | --- | --- | --- |
| Group | survival (%) (SE) | survival (%) (SE) | survival (%) (SE) | survival (%) (SE) | survival (%) (SE) |
| non-DM | 97.2 (0.6) | 91.5 (1.1) | 86.0 (1.4) | 80.7 (1.8) | 73.3 (2.4) |
| NIDDM | 96.5 (0.8) | 89.3 (1.5) | 82.5 (2.0) | 76.1 (2.5) | 67.3 (3.4) |
| IDDM | 98.0 (0.7) | 91.2 (1.7) | 83.1 (2.4) | 74.6 (2.9) | 62.1 (3.9) |

SE, standard error; non-DM, non-diabetes mellitus; NIDDM, non-insulin-dependent diabetes mellitus; IDDM, insulin-dependent diabetes mellitus
